# Supplementary material for: Autoreactive T-Cells in Psoriasis: Are They Spoiled Tregs and Can Therapies Restore Their Functions?
Source: Int J Mol Sci. 2023 Feb 22;24(5):4348. doi: 10.3390/ijms24054348 (PMC10002349; doi:10.3390/ijms24054348)
Supplement: Supplementary file 1 [file ijms-24-04348-s001.zip › ijms-2169206-supplementary.pdf]

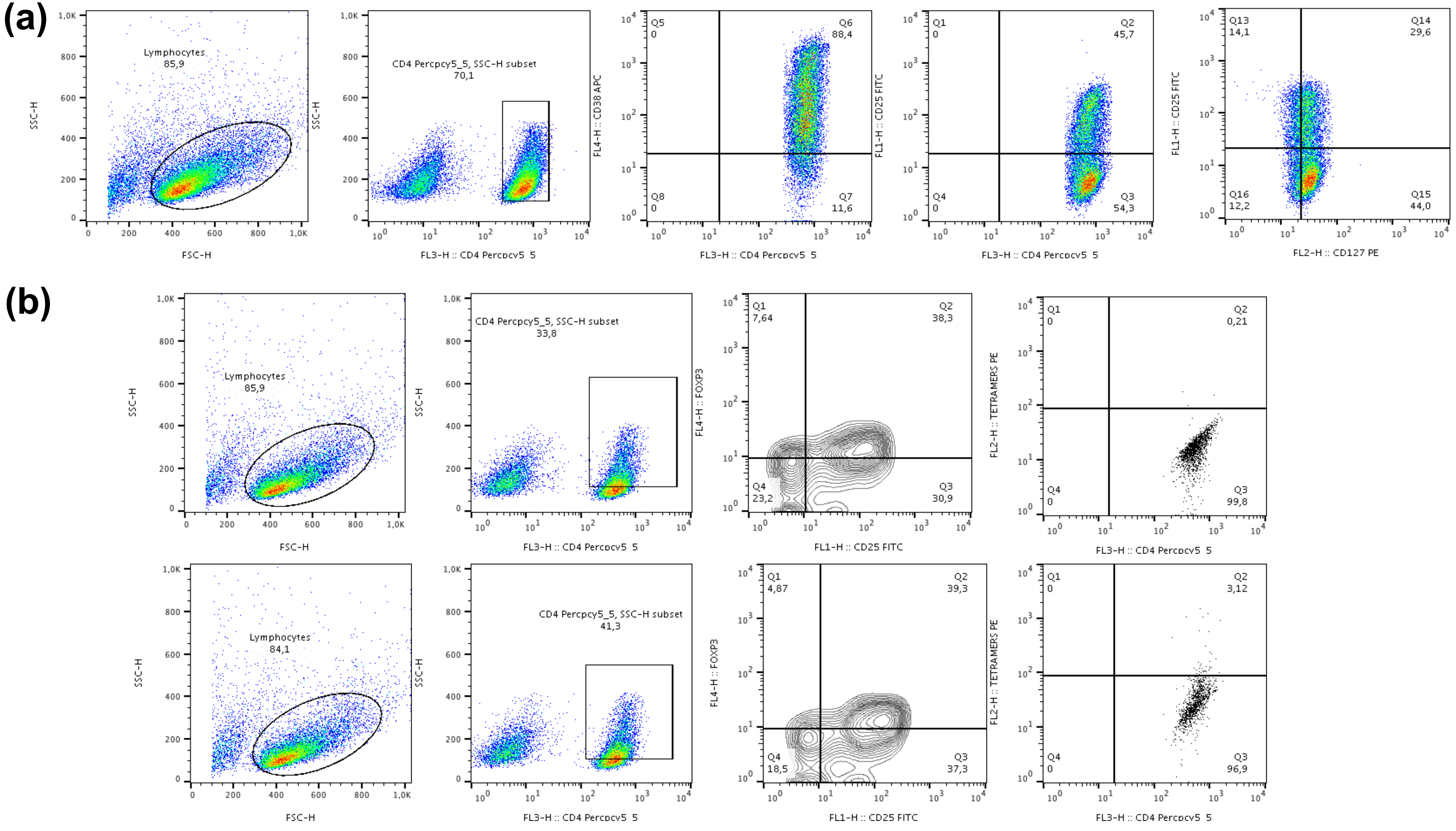

Figure S1. Phenotype of enriched Tregs isolated from PBMC. Tregs were isolated from PBMC from a HD and cultured for 3 weeks in the presence of 300U/mL of hrIL-2 **(a)**. The cells expressed high CD25 and CD38 and low CD127. **(b)** Intracellulare staining. for Foxp3 expression by the CD25^{high} cells, and expression of peptide-MHC-tetramers on the CD25^{high}Foxp3^{high} T-cells. Upper panel, staining for control peptide-MHC-tetramer, lower panel, staining for cognate peptide-MHC-tetramer (P6). Experiments were repeated three times, with the same donor.

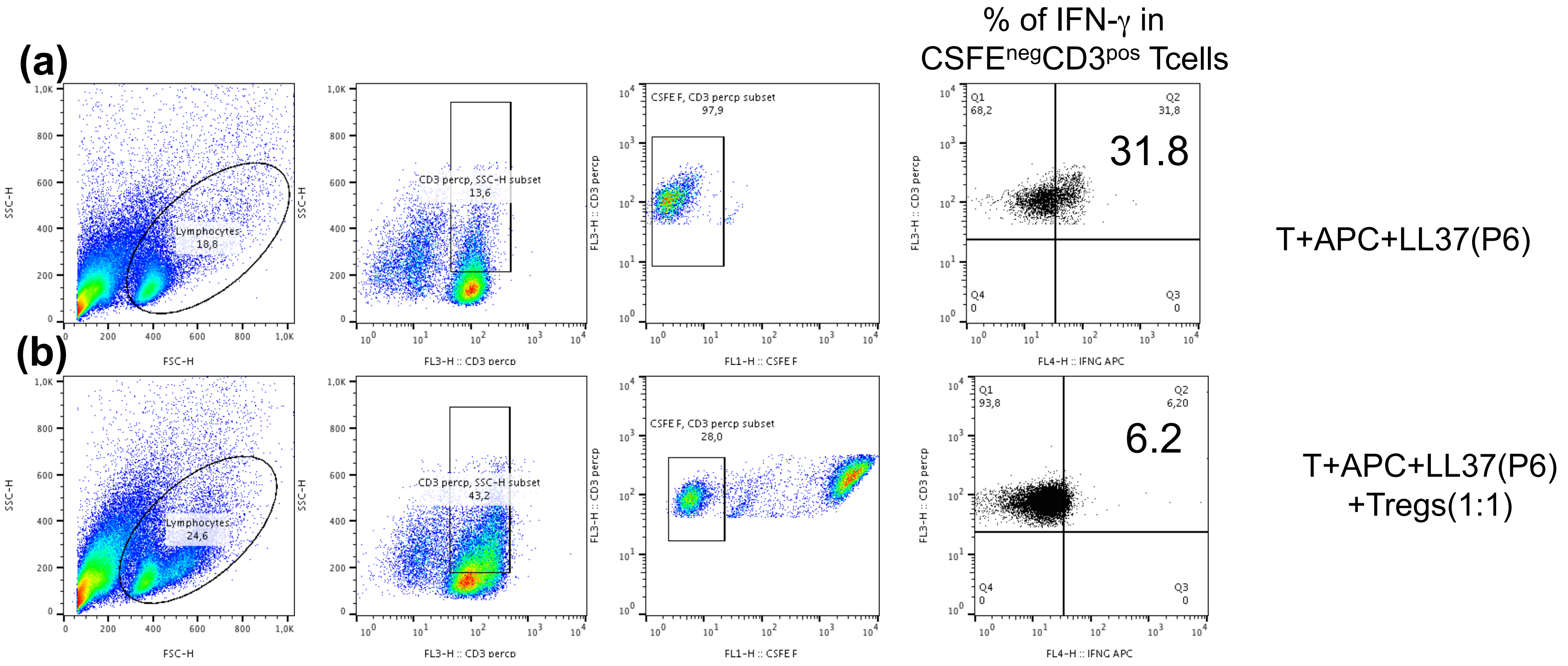

Figure S2. Gating strategy for T-cell suppression assays. A T-cell clone (T), derived from a HD and specific for LL37, was cultured either alone with APC presenting its cognate antigen LL37 P6 **(a)**, or **(b)**, in the presence of Tregs isolated and enriched from PBMC of the same HD (and pretreated with CSFE), also in the presence of APC and antigen (P6), over night. APC were irradiated to block their proliferation. The day after cells were harvested and IFN-γ production was assed, by intracellular staining in T-cells derived from T (which were CSFE negative and distinct from the CSFE^{+} Tregs) as described in Methods. Experiments were repeated two times (in duplicates), with the same donor.
